# Influence of Health Beliefs on COVID-19 Vaccination among Individuals with Cancer and Other Comorbidities in Puerto Rico

**DOI:** 10.3390/vaccines9090994

**Published:** 2021-09-06

**Authors:** McClaren Rodriguez, Andrea López-Cepero, Ana P. Ortiz-Martínez, Emma Fernández-Repollet, Cynthia M. Pérez

**Affiliations:** 1 Department of Epidemiology, Graduate School of Public Health, University of Pittsburgh, Pittsburgh, PA 15261, USA; mrr103@pitt.edu; 2 Department of Epidemiology, Rollins School of Public Health, Emory University, Atlanta, GA 30322, USA; 3 Division of Cancer Control and Population Sciences, University of Puerto Rico, Comprehensive Cancer Center, San Juan, PR 00936, USA; ana.ortiz7@upr.edu; 4 Department of Biostatistics and Epidemiology, Graduate School of Public Health, Medical Sciences Campus, University of Puerto Rico, San Juan, PR 00936, USA; cynthia.perez1@upr.edu; 5 Department of Pharmacology, Center for Collaborative Research in Health Disparities, School of Medicine, Medical Sciences Campus, University of Puerto Rico, San Juan, PR 00936, USA; e.fernandez@upr.edu

**Keywords:** COVID-19, vaccination, cancer, Puerto Rico, Health Belief Model

## Abstract

Ethnic minority populations are more likely to suffer from chronic comorbidities, making them more susceptible to the poor health outcomes associated with COVID-19 infection. Therefore, ensuring COVID-19 vaccination among vulnerable populations is of utmost importance. We aimed to investigate health behaviors and perceptions of COVID-19 vaccination among adults self-reporting diagnosis of cancer and of other chronic comorbidities in Puerto Rico (PR). This secondary analysis used data from 1911 participants who completed an online survey from December 2020 to February 2021. The Health Belief Model was used to measure perceptions surrounding COVID-19 vaccination among individuals self-reporting diagnosis of cancer and of other chronic comorbidities, and healthy adults. Among study participants, 76% were female, 34% were 50 years or older, 5% self-reported cancer diagnosis, and 70% had other chronic comorbidities. Participants self-reporting a cancer diagnosis had two times higher odds of getting vaccinated than healthy individuals (95% CI: 1.00–4.30). Compared to healthy participants, those self-reporting being diagnosed with cancer and those with chronic conditions other than cancer had significantly higher perceived COVID-19 susceptibility and severity. Our findings elucidate the effect of disease status on health-related decision-making and highlights information needed to be included in education campaigns to increase vaccine uptake among ethnic minority populations.

## 1. Introduction

Racial and ethnic health disparities continue to hamper prevention and treatment strategies of the coronavirus disease of 2019 (COVID-19) [1]. They experience increased morbidity from major chronic diseases, which are consequently associated with poorer health outcomes due to COVID-19 infection, including severe illness, hospitalization, and death [1]. Individuals in Puerto Rico (PR), a US territory comprised primarily of Hispanic individuals, have a high prevalence of acute and chronic medical conditions and the lowest percent of people reporting good/excellent health among all US states and territories [2,3]. The COVID-19 pandemic has worsened people’s health and social well-being living in PR, as it has compounded the financial crisis and catastrophic effects of previous natural disasters (hurricanes and seismic sequence) [4,5]. At the time of writing, 124,318 COVID-19 confirmed cases and 2562 deaths have been reported by the PR Department of Health [6]. Moreover, 59% of individuals in PR have been fully vaccinated against COVID-19 [7].

The presence of comorbidities is a significant risk factor for COVID-19 death [8,9,10]. A systematic review of 76 studies across 14 countries found that cancer patients, compared to patients with other comorbidities, have a higher risk of COVID-19 severe outcomes (admission to intensive care unit, invasive mechanical ventilation, and death) [11]. Another systematic review with over 63,000 COVID-19 patients showed a higher incidence of cancer than the general population and an increased risk of mortality among COVID-19 patients with cancer [12,13].

Due to the vulnerability of cancer patients, adherence to COVID-19 vaccination practices is critical. The immunocompromised state of cancer patients makes them exceedingly vulnerable to the effects of COVID-19; therefore, understanding their perceptions and choices regarding vaccination is essential for clinical applications. The Health Belief Model (HBM), a framework that has been used to understand individual health behaviors, including vaccination, revolves around constructs such as perceived susceptibility, severity, benefits, barriers to taking action, and cues to action [14,15,16,17]. Due to the increased risk of infection and mortality from COVID-19 among racial and ethnic minorities, especially those diagnosed with cancer, a comprehensive analysis of beliefs and attitudes about COVID-19 and vaccination against it is required to prevent further infection and the subsequent serious complications, including death.

Results from various studies showed that most cancer patients are willing to get the COVID-19 vaccine. The most common barriers to vaccine uptake among cancer patients were the fear of the vaccine’s side effects, efficacy, and safety. The strongest facilitator of vaccination was a clear explanation of the safety and efficacy of the vaccine from their physicians [18,19,20]. However, the current research has focused mainly on non-Hispanic White individuals and has not been explored in PR. The present study aims to fill this gap in research by identifying barriers and facilitators associated with COVID-19 vaccination among individuals self-reporting diagnoses of cancer and other comorbidities.

## 2. Materials and Methods

### 2.1. Study Design and Participants

This study was a secondary analysis that used data from a cross-sectional study that has been previously described [21]. The original study was conducted according to the guidelines of the Declaration of Helsinki and approved by the Institutional Review Board (IRB) of the University of Puerto Rico-Medical Sciences campus (IRB protocol number: 6050220). Briefly, the study was conducted in PR and collected data through convenience sampling, given the restrictions and limited resources during the pandemic. Data was collected through an anonymous web-based questionnaire. The online questionnaire was created using Google Forms and distributed to PR residents via institutional newsletters, academic groups, organizations, and social network pages. The web-based questionnaire was launched on 15 December 2020 and closed on 15 February 2021.

Eligible individuals were 18 years or older, residents of PR, and had access to an electronic device (i.e., computer, phone, or tablet) to complete the online questionnaire in Spanish. A total of 2233 surveys were submitted, of which, 21 responders declined to participate, 32 did not meet age and residency eligibility criteria, and 235 were duplicates. In all, 1945 responses were obtained. For the present analysis, individuals who reported a history of COVID-19 vaccination were excluded (*n* = 34), resulting in a final analytic sample of 1911 participants.

### 2.2. Study Measures

The online questionnaire collected data on vaccine intent, socio-demographic and clinical characteristics, health literacy, health behaviors, and beliefs surrounding COVID-19 infection and the vaccine.

#### 2.2.1. Disease Status

All participants responded to the question, “Has a doctor ever told you that you had or have any of the following health conditions?” A comprehensive list of over 30 health conditions was provided, including respiratory, psychiatric, endocrine, cardiovascular, cancer, autoimmune, rheumatic, neurological, kidney, and liver diseases. The participant was prompted to select “yes” if the health condition applied to them; otherwise, it was left blank and categorized as “no”. Individuals were further classified as “healthy” if no illness was reported, experiencing cancer if they self-reported a physician diagnosis of the disease, and with another comorbidity if they reported a physician diagnosis of any health conditions other than cancer.

#### 2.2.2. COVID-19 Vaccination Intent

Following previous studies [20,22], vaccination intent was measured with the question: “When a vaccine for the coronavirus becomes available, will you get vaccinated?” Response options were “Yes”, “No” and “Unsure”. Given the vaccine availability at the time (approved for emergency use in the US by the Food and Drug Administration on December 2020), a fourth option stating “I already got the vaccine” was added. Participants who responded “No” and “Unsure” were asked: “What makes you unwilling (or unsure) to get the vaccine?” Response options, adapted from earlier studies [15,18], were the following: Already got COVID-19, Safety, Efficacy, Cost, Lack of health insurance, Fear of needles, Not in a high-risk group, Do not trust what the government says about COVID-19, Novelty of the vaccine, Rigor of testing, Religious beliefs, and Other (which were then specified in a text field). The intent to get vaccinated was evaluated with the three possible response options (yes, no, and unsure) for descriptive purposes.

#### 2.2.3. COVID-19 Beliefs and Vaccination against It

HBM constructs were used to evaluate COVID-19 beliefs, vaccination hesitancy, and unwillingness as in Wong et al. [15]. Survey questions measured perceived COVID-19 susceptibility (i.e., My chance of getting COVID-19 in the next few months is high), severity (i.e., The complications from contracting COVID-19 are serious), perceived benefits towards COVID-19 vaccination (i.e., Vaccination decreases my chances of getting COVID-19 or its complications), cues to action for vaccination (i.e., I will only take the COVID-19 vaccine if I was given adequate information about it), and barriers for COVID-19 vaccination (i.e., I am concerned about the efficacy of the COVID-19 vaccination). As in Wong et al., response options to all previously described items were “Agree” or “Disagree” [15].

#### 2.2.4. COVID-19 Experience across Socio-Demographic, Behavioral, and Clinical Characteristics

Socio-demographic characteristic data included: age, sex, education, annual household income, and employment status. Data was also collected regarding whether the participant received the influenza vaccine in the previous year. Religiosity was evaluated by asking about the importance of religion in the person’s life (response options ranging from less important to very important) [18]. Health literacy was assessed by asking about the participant’s confidence in filling out medical forms by themselves (range of response options: extremely uncomfortable to extremely comfortable) [23].

### 2.3. Statistical Analysis

Chi-square or Fisher’s exact tests were used to assess the associations between each HBM construct and disease status. A logistic regression model was built to determine the odds of willingness to get vaccinated against by disease status, with “no or unsure” and “healthy” groups used as the reference. We also modeled each HBM construct by disease status. The regression models were adjusted for age, sex, education, income, influenza vaccine, employment status, religiosity, and confidence in filling out medical records (health literacy), as done in earlier studies [18,22]. Model fit was assessed using Hosmer and Lemeshow’s goodness-of-fit test. To determine if there were any problems with multicollinearity between the independent variables, variance inflation factors and tolerance were evaluated. Odds ratios (OR) and corresponding 95% confidence intervals (CI) were calculated to report results from the regression models. Statistical analyses were performed using STATA for Macintosh release 16.1 (StataCorp L.P., College Station, TX, USA).

## 3. Results

Sample characteristics are shown by disease status in Table 1. Overall, most participants were females (76.2%, *n* = 1444) and employed (65.7%, *n* = 1256) (Table 1). Over 80% of participants had received an undergraduate degree or higher, and approximately 75% reported having an annual income greater than $20,000. In general, around half of the participants had received the influenza vaccine in the past year (50.6%, *n* = 966), and 45.2% (*n* = 864) felt extremely comfortable filling out medical forms. Out of the total sample, 70% (*n* = 1338) reported diagnoses of comorbidities other than cancer, 5.1% (*n* = 97) reported a cancer diagnosis, and 24.9% (*n* = 476) were healthy. Compared to healthy individuals and those self-reporting being diagnosed with other comorbidities, those self-reporting cancer diagnosis were more likely to be over 50 years old (*p* < 0.001) and report that religiosity was very important to them (*p* = 0.003).

In a bivariate analysis, vaccine intent was high across all groups (82.5%). Individuals in the cancer group were more likely to have high perceived susceptibility to COVID-19, and healthy individuals were more likely to not perceive themselves as susceptible to COVID-19 (Table 2). A greater proportion of individuals self-reporting a cancer diagnosis (61.9%, *n* = 60), followed by those self-reporting diagnoses of other comorbidities (59.1%, *n* = 791), agreed that their chances of getting COVID-19 were high as compared to a lower proportion of healthy individuals (*p* = 0.006) (Table 2). Individuals self-reporting a cancer diagnosis were also more worried about their likelihood of getting COVID-19 (*p* = 0.002) and believed that getting COVID-19 was currently a possibility for them (*p* = 0.007). Furthermore, the aforementioned group was more likely to perceive they would get very sick if infected with COVID-19 (*p* < 0.001) and were afraid of COVID-19 infection (*p* < 0.001). However, individuals self-reporting a cancer diagnosis were less worried about the side-effects of COVID-19 vaccination (70.1%, *n* = 68) than individuals self-reporting diagnoses of comorbidities other than cancer (59.5%, *n* = 796) and healthy individuals (66.0%, *n* = 314; *p* = 0.009). Lastly, individuals self-reporting comorbidities other than cancer were more likely to take the vaccine if given adequate information about it than those self-reporting a cancer diagnosis and healthy individuals (*p* = 0.003) (Table 2).

Similar results were observed after adjusting for covariates (Table 3). Individuals self-reporting a cancer diagnosis were two times more likely to want to get the COVID-19 vaccine once it was available to them than healthy individuals (95% CI: 1.00–4.30). Higher odds of perceived susceptibility to COVID-19 were found for both individuals self-reporting a cancer diagnosis (OR: 1.63, 95% CI: 1.10–2.62) and other comorbidities (OR: 1.39, 95% CI: 1.11–1.73) compared to healthy individuals. Participants self-reporting a cancer diagnosis had ten times higher odds of reporting being worried about their likelihood of getting COVID-19 (*p* = 0.026); however, the sample size was too small to produce a reliable estimate. We also found an association between individuals who self-reported having diagnoses of comorbidities other than cancer and being worried about the likelihood of getting COVID-19 (OR: 1.63, 95% CI: 1.09–2.44). Individuals in the cancer group and those in the other comorbidities group were both more likely to perceive that getting COVID-19 was currently a possibility for them, compared to healthy participants (OR: 1.94, 95% CI: 1.16–3.25; OR: 1.56, 95% CI: 1.24–1.97; respectively). Those in the cancer group had four times higher odds of perceiving they would get very sick if they contracted COVID-19 than healthy individuals (95% CI: 2.30–7.58). Similarly, those in the other comorbidities group were two times more likely to perceive that they would get very sick if they contracted COVID-19 compared to healthy participants (95% CI: 1.47–2.28). Additionally, participants who self-reported a diagnosis of cancer and those with comorbidities other than cancer had higher odds of being afraid of COVID-19 infection compared to healthy individuals (OR: 2.58, 95% CI: 1.18–5.35; OR: 1.67, 95% CI: 1.25–2.22; respectively). Those self-reporting diagnoses of comorbidities other than cancer were 1.42 times more likely to take the COVID-19 vaccine if they were given adequate information about the vaccine than healthy participants (95% CI 1.14–1.77).

We also investigated the reasons for vaccine hesitancy. Vaccine safety (11.2%) was the main reason for COVID-19 vaccine hesitancy, as reported by 13% of healthy participants, 6.2% of individuals self-reporting a cancer diagnosis, and 10.8% of those self-reporting diagnoses of comorbidities other than cancer. The efficacy (8.6%) and novelty (8%) of the COVID-19 vaccine were also leading factors for vaccine hesitancy among participants (data not shown).

## 4. Discussion

The present study showed that COVID-19 vaccine intent varied by disease status. Respondents self-reporting a cancer diagnosis or diagnoses with other comorbidities other than cancer were more likely to report intent to receive the COVID-19 vaccine compared to healthy individuals, possibly due to our results showing high perceived severity and susceptibility among these participants.

Approximately 49% of the total population in the US is fully vaccinated, compared to 59% in PR [7,24]. Currently, PR is among US states and territories with the highest number of doses administered per 100,000 people [24], showing substantial improvement in vaccination practices in the archipelago compared to previous influenza epidemics [25]. It is also important to note that the COVID-19 vaccine was made available for adults 65 years or older in PR on 2 February 2021, whereas the vaccine was not available to residents in PR with comorbidities including cancer, chronic renal disease, pulmonary disease, fibrosis, Down syndrome, heart disease, and immunocompromised individuals until 11 March 2021 [26].

In this study, participants self-reporting a cancer diagnosis were more likely to report an intent to get vaccinated, with 88% indicating they would get the COVID-19 vaccine when it was made available. Other studies published similar results regarding positive uptake of the COVID-19 vaccine among individuals with cancer [18,19,20,27]. For example, a study evaluating COVID-19 vaccine hesitancy among individuals with comorbidities reported that over 80% of individuals with cancer, undergoing or not undergoing treatment at the time, would receive the vaccine [27]. Adversely, it is also important to contemplate the effect of cancer on vaccine efficacy and immune response. Cancer patients with active malignancies were excluded from COVID-19 vaccine clinical trials [28,29,30]. Individuals with cancer were then underrepresented in the study samples, creating a paradox where this high-risk group should have been prioritized for immunization. Still, not enough evidence was available to inoculate individuals with cancer. Since then, recommendations have been updated to support vaccination for all individuals with cancer.

Issues continue to burden individuals with cancer undergoing immunosuppressive anticancer therapies where the vaccine’s efficacy may be inhibited, continuing to leave these patients unprotected against COVID-19 illness [28]. At the time the present survey was conducted, less was known about the impact of the severe acute respiratory syndrome coronavirus 2 (SARS-CoV-2) antibody development in cancer patients. Considering this, a recent review of the literature showed mixed findings about vaccine efficacy in cancer patients. One study reported suboptimal results, where almost half of the cancer patients in the study (46.3%) did not develop antibodies against the SARS-CoV-2 virus [30]. Parallel studies that evaluated the antibody response to mRNA vaccines in cancer patients revealed promising results, with 90–94% of cancer patients in the study samples developing antibodies [31,32]. However, a commonality among these studies was that cancer patients actively undergoing immunosuppressive treatment were more likely to have a lower antibody response from the COVID-19 vaccine. Therefore, even though individuals self-reporting a cancer diagnosis in our study had a high vaccine intent, additional research is needed to understand the magnitude and duration of COVID-19 vaccine-induced immunity in cancer patients. Healthy individuals should continue to follow COVID-19 prevention recommendations to protect cancer patients, especially those undergoing treatment for which the COVID-19 vaccine is much less effective, to lower the risk of COVID-19 transmission and infection in this population.

We found that vaccine safety was the most reported barrier to COVID-19 vaccine uptake among vaccine-hesitant participants. Concerns over vaccine safety, efficacy, and side effects were not significantly associated with disease status. Other studies have reported that vaccine safety, efficacy, and novelty are significant reasons for vaccine refusal [20,21,27,33,34]. A cross-sectional study that evaluated vaccine perceptions among high-risk adults in the UK found that participants who did not want to get the vaccine were worried about the fast development and efficacy of the vaccine [33]. Addressing vaccine safety as a common barrier to COVID-19 vaccination among individuals with cancer and those with other comorbidities will be imperative for future vaccination campaigns.

A self-reported diagnosis of cancer and chronic comorbidities other than cancer was associated with high perceived disease susceptibility. Significant associations were also found between disease status and perceived disease severity, barriers to vaccination, and cues to action. These results coincide with several other studies that evaluated health decisions regarding the COVID-19 vaccine among individuals with chronic comorbidities [18,27,33,34]. Lou et al. reported that individuals who were either actively treated or had a history of cancer were significantly more likely to report high levels of concern regarding COVID-19 infection compared to those with no history of cancer [35]. The results from a survey distributed to 804 US adults were also analogous with ours, where perceived severity of COVID-19 infection among people with pre-existing conditions significantly predicted vaccination [34]. In a US study conducted by Kelkar et al., empathy played a role in vaccine willingness among cancer patients [20]. Disease status affects health behaviors; people who have experienced cancer or other comorbidities tend to prioritize their health and the health of others more than those who are generally healthy [20,35]. The high perceived risk among individuals self-reporting cancer and other comorbidities in our study amplifies the need for effective risk communication in healthcare settings. For example, studies have shown that when physicians explained the effects and benefits of vaccination to cancer patients, they are more likely to adopt this preventative behavior [20,35,36]. Healthcare systems in PR can use these results to update and inform physician recommendations for COVID-19 vaccination among individuals with cancer and those with other chronic conditions.

The results from this study should be interpreted with strengths and limitations in mind. A strength of the current research is using the HBM model to guide survey development and data analysis. The HBM is a notable model for conceptualizing health behaviors, including COVID-19 vaccine intent, as seen in other studies [15,16,17]. Our findings were novel due to the use of the HBM model to identify COVID-19 vaccine barriers and facilitators specifically among individuals with chronic comorbidities and cancer, as well as a primarily Hispanic study population. Also, the study provides essential information on beliefs about COVID-19 and vaccination against it for at-risk individuals in PR. However, a study limitation is that the questionnaire was distributed online, therefore, individuals without access to an electronic device did not access the survey. This implication resulted in a higher proportion of participants who were women and individuals of higher education income, which could have introduced selection bias and a lack of generalizability of the study results; this is common for studies relying on online surveys for data collection [37]. In addition, given that the online questionnaire was anonymous, we were not able to confirm disease diagnosis with medical health records or by contacting healthcare providers; however, disease status was assessed with an item that specifically asked about diagnosis. Our study also lacked specific information on the treatment status of individuals who reported cancer. This can lead to misclassification of vaccine intent outcomes [18,27,35]. Additionally, data on type of cancer and prognosis were not originally collected in the study; thus, future studies need to take these factors into consideration. Lastly, the HBM assumes that there is equal distribution of information on COVID-19. Future studies should explore equitable access to information on COVID-19 in high-risk population subgroups.

## 5. Conclusions

This study showed that individuals self-reporting cancer and other comorbidities other than cancer had high COVID-19 vaccination intent and perceived their risk to the effects of COVID-19 illness as high. It remains vital to continue to identify barriers and facilitators of vaccination and monitor vaccine uptake among individuals with cancer and other comorbidities. Our results provide constructive discourse on the topic of COVID-19 vaccination among high-risk and healthy Hispanic individuals in PR. Future health policies and vaccination programs in PR should include specific recommendations for high-risk individuals to increase vaccination uptake and prioritize those at higher risk of severe complications from COVID-19 infection.

## Figures and Tables

**Table 1:** Descriptive characteristics of study sample by disease status among adults living in PR (*n* = 1911).

	Disease Status				
Characteristic	Total Sample *n* = 1911 *n* (%)	Healthy *n* = 476 (24.9%)	Cancer *n* = 97 (5.1%)	Other Comorbidities *n* = 1338 (70.0%)	*p*-Value
Sex ^1^					0.258
Female Male	1444 (76.2) 451 (23.8)	348 (73.6) 125 (26.4)	71(74.7) 24 (25.3)	1025 (77.3) 302 (22.7)	
Age group (years)					<0.001
18–29	481 (25.2)	168 (35.3)	3 (3.1)	310 (23.2)	
30–39	361 (18.9)	101 (21.2)	15 (15.5)	245 (18.3)	
40–49	426 (22.3)	118 (24.8)	10 (10.3)	298 (22.3)	
≥50	643 (33.6)	89 (18.7)	69 (71.1)	485 (36.2)	
Highest education level					0.710
High school graduate or less	70 (3.6)	16 (3.4)	4 (4.1)	50 (3.8)	
Associate degree	168 (8.8)	50 (10.5)	7 (7.2)	111 (8.3)	
Some college	101 (5.3)	19 (4.0)	7 (7.2)	75 (5.6)	
Undergraduate degree	691 (36.2)	183 (38.4)	37 (38.1)	471 (35.2)	
Masters	537 (28.1)	128 (26.9)	26 (26.9)	383 (28.6)	
Doctoral degree	344 (18.0)	80 (16.8)	16 (16.5)	248 (18.5)	
Employment status					0.062
Working	1256 (65.7)	316 (66.4)	53 (54.6)	887 (66.3)	
Not working	655 (34.3)	160 (33.6)	44 (45.4)	451 (33.7)	
Annual household income					0.321
≤$20,000	325 (17.0)	75 (15.8)	22 (22.7)	228 (17.0)	
$20,001–$40,000	474 (24.8)	109 (22.9)	21 (21.6)	344 (25.7)	
$40,001–$75,000	461 (24.1)	119 (25.0)	22 (22.7)	320 (23.9)	
>$75,000	466 (24.4)	129 (27.1)	27 (27.8)	310 (23.2)	
Prefer not to answer	185 (9.7)	44 (9.2)	5 (5.2)	136 (10.2)	
Received influenza vaccine in the previous year					0.053
Yes	966 (50.5)	222 (46.6)	57 (58.8)	687 (51.3)	
No	945 (49.5)	254 (53.4)	40 (41.2)	651 (48.7)	
Importance of religiosity					0.003
Less important	411 (21.5)	112 (23.5)	13 (13.4)	286 (21.4)	
Somewhat important	338 (17.7)	96 (20.1)	14 (14.4)	228 (17.0)	
Important	498 (26.1)	134 (28.2)	23 (23.7)	341 (25.5)	
Very important	664 (34.7)	134 (28.2)	47 (48.5)	482 (36.1)	
Confidence filling out medical forms					0.255
Extremely/a little uncomfortable	99 (5.2)	19 (4.0)	5 (5.2)	75 (5.6)	
Neutral	404 (21.1)	85 (17.9)	18 (18.6)	301 (22.5)	
Very comfortable	544 (28.5)	141 (29.6)	28 (28.9)	375 (28.0)	
Extremely comfortable	864 (45.2)	231 (48.5)	46 (47.3)	587 (43.9)	

^1^ 16 participants reported “prefer not to answer”. Column percentages are shown.

**Table 2:** Bivariate association between vaccine hesitancy predictors and disease status among adults in PR.

	Disease Status			
Characteristic	Healthy	Cancer	Other Comorbidities	*p*-Value
**Vaccine Intent**				
Do you have plans to get vaccinated against COVID-19 when a vaccine is available?				0.093
Yes	381 (80.0)	86 (88.7)	1110 (83.0)	
No or unsure	95 (20.0)	11 (11.3)	228 (17.0)	
**Perceived susceptibility**				
My chance of getting COVID-19 in the next few months is high				0.006
Agree	243 (51.0)	60 (61.9)	791 (59.1)	
Disagree	233 (49.0)	37 (38.1)	547 (40.9)	
I am worried about the likelihood of getting COVID-19				0.002
Agree	431 (90.5)	96 (99.0)	1257 (94.0)	
Disagree	45 (9.5)	1 (1.0)	81 (6.0)	
Getting COVID-19 is currently a possibility for me				0.007
Agree	300 (63.0)	70 (72.2)	945 (70.6)	
Disagree	176 (37.0)	27 (27.8)	393 (29.4)	
**Perceived severity**				
The complications from contracting COVID-19 are serious				0.359
Agree	458 (96.2)	96 (99.0)	1300 (97.2)	
Disagree	18 (3.8)	1 (1.0)	38 (2.8)	
I will be very sick if I get COVID-19				<0.001
Agree	236 (49.6)	82 (84.5)	894 (66.8)	
Disagree	240 (50.4)	15 (15.5)	444 (33.2)	
I am afraid of getting COVID-19				<0.001
Agree	379 (79.6)	88 (90.7)	1160 (86.7)	
Disagree	97 (20.4)	9 (9.3)	178 (13.3)	
**Perceived benefits**				
Vaccination is a good idea because it makes me feel less worried about catching COVID-19				0.174
Agree	384 (80.7)	84 (86.6)	1123 (83.9)	
Disagree	92 (19.3)	13 (13.4)	215 (16.1)	
Vaccination decreases my chances of getting COVID-19 or its complications				0.156
Agree	411 (86.3)	90 (92.8)	1185 (88.6)	
Disagree	65 (13.7)	7 (7.2)	153 (11.4)	
**Perceived barriers**				
I worry the possible side-effects of COVID-19 vaccination would interfere with my usual activities				0.009
Agree	162 (34.0)	29 (29.9)	542 (40.5)	
Disagree	314 (66.0)	68 (70.1)	796 (59.5)	
I am concerned about the efficacy of the COVID-19 vaccination				0.493
Agree	190 (39.9)	41 (42.3)	576 (43.1)	
Disagree	286 (60.1)	56 (57.7)	762 (56.9)	
I am concerned about the safety of the COVID-19 vaccination				0.597
Agree	184 (38.7)	42 (43.3)	546 (40.8)	
Disagree	292 (61.3)	55 (56.7)	792 (59.2)	
**Cues to action**				
I will only take the COVID-19 vaccine if I am given adequate information about it				0.003
Agree	260 (54.6)	54 (55.7)	845 (63.2)	
Disagree	216 (45.4)	43 (44.3)	493 (36.8)	
I will only take the COVID-19 vaccine if the vaccine is taken by many in the public				0.144
Agree	77 (16.2)	20 (20.6)	271 (20.2)	
Disagree	399 (83.8)	77 (79.4)	1067 (79.8)	

Column percentages are shown.

**Table 3:** COVID-19 vaccine intent and predictors by disease status using logistic regression.

	Cancer Diagnosis		Other Comorbidities	
Outcomes	Crude OR (95% CI)	Adjusted OR (95% CI)	Crude OR (95% CI)	Adjusted OR (95% CI)
**Vaccine Intent**				
Do you have plans to get vaccinated against COVID-19 when a vaccine is available?				
Yes	1.95 (1.00–3.79)	2.08 (1.00–4.30) *	1.21 (0.93–1.58)	1.29 (0.96–1.73)
No or unsure	1.00	1.00	1.00	1.00
**Perceived Susceptibility**				
My chance of getting COVID-19 in the next few months is high				
Agree	1.55 (0.99–2.43)	1.63 (1.01–2.62) *	1.39 (1.12–1.71)	1.39 (1.11–1.73) *
Disagree	1.00	1.00	1.00	1.00
I am worried about the likelihood of getting COVID-19				
Agree	10.02 (1.36–73.6)	10.10 (1.32–77.2)	1.62 (1.11–2.37)	1.63 (1.09–2.44) *
Disagree	1.00	1.00	1.00	1.00
Getting COVID-19 is currently a possibility for me				
Agree	1.52 (0.94–2.46)	1.94 (1.16–3.25) *	1.41 (1.13–1.76)	1.56 (1.24–1.97) *
Disagree	1.00	1.00	1.00	1.00
**Perceived Severity**				
The complications from contracting COVID-19 are serious				
Agree	3.77 (0.50–28.6)	3.92 (0.48–31.4)	1.34 (0.76–2.38)	1.40 (0.77–2.55)
Disagree	1.00	1.00	1.00	1.00
I will be very sick if I get COVID-19				
Agree	5.56 (3.12–9.92)	4.18 (2.30–7.58) *	2.05 (1.66–2.53)	1.83 (1.47–2.28) *
Disagree	1.00	1.00	1.00	1.00
I am afraid of getting COVID-19				
Agree	2.50 (1.22–5.15)	2.51 (1.18–5.35) *	1.67 (1.27–2.19)	1.67 (1.25–2.22) *
Disagree	1.00	1.00	1.00	1.00
**Perceived Benefits**				
Vaccination is a good idea because it makes me feel less worried about catching COVID-19				
Agree	1.55 (0.83–2.90)	1.57 (0.80–3.06)	1.25 (0.96–1.64)	1.32 (0.99–1.76)
Disagree	1.00	1.00	1.00	1.00
Vaccination decreases my chances of getting COVID-19 or its complications				
Agree	2.03 (0.90–4.58)	2.30 (0.97–5.45)	1.22 (0.89–1.67)	1.31 (0.93–1.83)
Disagree	1.00	1.00	1.00	1.00
**Perceived Barriers**				
I worry the possible side-effects of COVID-19 vaccination would interfere with my usual activities				
Agree	0.83 (0.51–1.33)	0.69 (0.41–1.16)	1.32 (1.06–1.64)	1.23 (0.97–1.56)
Disagree	1.00	1.00	1.00	1.00
I am concerned about the safety of the COVID-19 vaccination				
Agree	1.21 (0.79–1.89)	1.08 (0.67–1.76)	1.09 (0.88–1.36)	0.99 (0.79–1.26)
Disagree	1.00	1.00	1.00	1.00
I am concerned about the efficacy of the COVID-19 vaccination				
Agree	1.10 (0.71–1.72)	1.02 (0.63–1.65)	1.14 (0.92–1.41)	1.04 (0.82–1.31)
Disagree	1.00	1.00	1.00	1.00
**Cues to Action**				
I will only take the COVID-19 vaccine if I was given adequate information about it				
Agree	1.00	1.00	1.00	1.00
Disagree	1.04 (0.67–1.62)	1.14 (0.72–1.81)	1.42 (1.15–1.76)	1.42 (1.14–1.77) *
I will only take the COVID-19 vaccine if the vaccine is taken by many in the public				
Agree	1.00	1.00	1.00	1.00
Disagree	1.35 (0.78–2.33)	1.22 (0.68–2.19)	1.32 (0.99–1.74)	1.23 (0.92–1.65)

Adjusted for influenza vaccine, health literacy, religiosity, age, sex, education, income, and employment status. * *p* < 0.05.

## Data Availability

Derived data supporting the findings of this study are available from the corresponding author upon request.

## Abbreviations

Confidence interval (CI), Coronavirus disease of 2019 (COVID-19), Health Belief Model (HBM), odds ratio (OR), Puerto Rico (PR), and severe acute respiratory syndrome coronavirus 2 (SARS-CoV-2).
